# Organ at risk sparing by non-coplanar prone breast radiotherapy on Halcyon/Ethos linacs utilizing breast couch slewing

**DOI:** 10.1038/s41598-026-54571-4

**Published:** 2026-06-08

**Authors:** Bruno Speleers, Michael E. J. Stouthandel, Vincent Vakaet, Leen Paelinck, Max Schoepen, Annick Van Greveling, Ann Van Esch, Anne-Catherine Wéra, Vincent Remouchamps, Tom Van Hoof, Liv Veldeman, Werner De Gersem, Wilfried De Neve

**Affiliations:** 1 Department of Human Structure and Repair, Ghent University, C. Heymanslaan 10, Radiotherapy park, entrance 98, B-9000 Ghent, Belgium; 2 Cancer Research Institute Ghent (CRIG), Ghent, Belgium; 3 Department of Radiation Oncology, Ghent University Hospital, C. Heymanslaan 10, Radiotherapy park, entrance 98, B-9000 Ghent, Belgium; 4 Hydraulic engineering laboratory, Flanders Hydraulics, Berchemlei 115, B-2140 Antwerp, Belgium; 5 7Sigma Radiotherapy Physics, Kasteeldreef 2, B-1350 Tildonk, Belgium; 6 Department of Radiotherapy, CHU-UCL Namur, Site Ste Elisabeth, Place Louise Godin 15, B-5000 Namur, Belgium

**Keywords:** Radiotherapy, Breast cancer, Treatment planning, Organ at risk sparing, Prone position, Deep inspiration breath hold, Cancer, Medical research, Oncology, Physics

## Abstract

Organ at risk (OAR)-dose reductions are needed to minimize radiation toxicity in settings with high OAR-doses, like adjuvant breast and lymph node radiotherapy. Non-coplanar techniques, combined with prone positioning and deep inspiration breath hold (DIBH) techniques, could accomplish this. A planning study was performed in patients requiring left-side adjuvant breast and axillary/periclavicular lymph node irradiation (*n* = 8). Simulation was performed in prone crawl position using DIBH. Coplanar and non-coplanar short-arc VMAT plans were compared. Non-coplanar plans featured ≤ 20° angular separation between planes. Feasibility was tested using a CAD model, including a prone crawl breast couch positioned in an up to 20° angle (transverse plane) with the couchtop. Non-coplanar techniques can further reduce radiation exposure. Adding beams in planes with ± 15° to ± 20° angular separation with the transverse plane yielded > 20% mean-dose reductions simultaneously to heart, lungs and esophagus, compared to coplanar plans. Plans with ≤ ± 20° angular separation with the transverse plane proved feasible on linacs lacking couch isocenter rotations. Angular separation of ± 15° seems the best compromise between OAR-sparing and technical challenges. Non-coplanar radiotherapy options yielded superior OAR sparing compared to coplanar techniques in patients requiring adjuvant breast and lymph node irradiation and should be considered for improved radiation toxicity prevention.

## Introduction

Reduction of breast cancer-specific mortality by radiotherapy after breast-conserving surgery is counter-acted by dose-dependent excess mortality, mainly from radiation induced cardiac injury and lung cancer^1–4^. The deep inspiration breath hold (DIBH) technique, instead of free breathing, can be used to reduce the dose on the heart and lungs, as a result of a more favorable anatomical position of the heart during DIBH. An additional benefit of deep inspiration breath holds is the reduction of organ at risk movement during treatment^5^. Dose to lungs and heart can also be decreased by treating patients in prone position instead of supine position^6^. Prone positioning involves the use of a prone breast board or couch that consists of a support surface and a breast aperture. By gravity, the breast is pulled through the aperture, away from lungs and heart. The breast narrows in the direction of irradiation and skin folds are stretched. These anatomical changes may explain the significantly reduced acute toxicity and cosmetic changes observed in randomized trials comparing prone and supine radiotherapy^7–9^. Level I evidence of reduced toxicity in prone radiotherapy has been generated in the setting of whole breast treatment using coplanar radiation techniques^7,9^. Lower levels of evidence have been generated in other settings including partial breast irradiation^10,11^ and irradiation of the breast and regional lymph nodes^12–14^. Reduction of lung dose and a variable effect on heart dose in prone treated patients has been reported in studies comparing prone and supine positions in breast and regional lymph node radiotherapy. DIBH can be combined with prone position, resulting in additional OAR sparing effect^15,16^ and this combination was recently listed as the most promising approach for breast cancer treatment in the future^17^. The treatment of breast and regional lymph nodes via the use of coplanar beam arrangements resulted in decreased radiation doses to ipsilateral and contralateral lung, thyroid, contralateral breast and esophagus, as compared to radiotherapy in supine position^12^. There were no significant differences for heart and humeral head doses. However, lower levels of radiation doses to OARs seemed achievable by using non-coplanar beam directions^14^, but direct comparisons of coplanar versus non-coplanar were non-existent. In Pubmed, the keywords ‘breast cancer radiotherapy’ & ‘prone position’ & ‘lymph node irradiation’ yield 25 publications, none of which report on direct comparison of coplanar versus non-coplanar beam set-ups.

Most prone breast couches are designed to support the patient in an anatomical position with both arms elevated, further referred to as the ‘prone dive’ position. The prone dive position is well suited for coplanar irradiation of whole breast. However, in breast and lymph node radiotherapy, beam access is restricted by the elevated arms and the hardware parts of prone dive breast couches underneath the shoulder and arm at the side to be irradiated. Our research group has developed the prone crawl breast couch (PCBC), which expanded the range of beam access to the lymph node regions as compared to prone dive breast couches. In ‘prone crawl’, the ipsilateral arm (arm on the side to be irradiated) is placed along the body and the contralateral arm above the head. The ipsilateral arm is supported underneath by a narrow carbon fiber composite blade. No support structure obstructs beam access to the axillary, periclavicular, or internal mammary lymph node chains for a 0° to |70°| range of couch isocenter rotations. In previous studies, the non-coplanar dose distributions were obtained using couch isocenter rotations of ± 45° to ± 70°^14^. Couch isocenter and tabletop rotations are not available on a popular new class of linear accelerators with open (Elekta Harmony), or enclosed (Varian Halcyon and Ethos) gantries. These accelerators focus on coplanar treatments with all beam axes in the transverse plane. Beams with small angles with the transverse plane are theoretically possible by slewing or tilting a patient support system, like for example the PCBC, against the tabletop of the linear accelerator.

Slewing is the rotation of the PCBC relative to the tabletop around the z-axis thereby changing the angle between the X-Y coordinate systems of PCBC and tabletop (the slewing angle).

The first aim of this study was to compare coplanar with non-coplanar dose distributions in the setting of radiotherapy of whole breast, integrated boost and levels 2, 3 and 4 lymphatic target volumes. Should non-coplanar dose distributions prove beneficial in this setting, the technical feasibility/possibilities on currently available linear accelerators should also be explored.

As such, the second aim of this study was to investigate the maximum angular separation with the transverse plane that could be obtained by slewing the PCBC against the tabletop inside the cylindrical gantry of a Varian Halcyon/Ethos linear accelerator.

## Patients and methods

CT-anatomical data to perform in-silico treatment simulations were obtained from a previous trial approved by the Ghent University Hospital Ethics Board (reference number: EC-UZ-2016/0351, Belgian Registration Number: B670201628048). All CT acquisitions were performed using 0° slewing angle. Slewing angles for treatment were simulated in the virtual world using 3D-transforms. All experimental protocols were approved by the Ghent University Hospital Ethics Board and all patients signed informed consent. All methods were carried out in accordance with the 1964 declaration of Helsinki and all subsequent revisions and according to the institutional guidelines and regulations.

Eight patients with left-sided breast cancer were simulated during a short deep inspiration breath hold (DIBH) on a prone crawl breast couch (PCBC) as described previously^18^. The number of patients included in the current study was chosen for hypothesis generation and investigating feasibility. If the results are promising, larger cohorts can be included in a follow up study. The PCBC was specifically designed for whole breast and regional lymph node irradiation. The device is described elsewhere and yields lower doses to OARs while maintaining target coverage when compared to supine whole breast and regional lymph node irradiation^12–14^. Delineation of clinical target volumes (CTV) of whole breast and tumor region was based on guidelines proposed by the ESTRO consensus^19,20^. Delineation of the lymphatic CTV was based on the prone crawl specific guidelines by Stouthandel et al.^21^. The lymphatic CTV included lymph node regions 2, 3 and 4. Organs-at-risk (OAR) were lung, heart, left anterior descending coronary artery (LAD), contralateral breast, esophagus, spinal cord, thyroid and brachial plexus. A dose of 40.05 Gy (Gy) in 15 fractions was planned for the whole breast and lymphatic PTV with an integrated boost of 15 × 3.33 Gy to the CTV of the tumor bed. The high boost dose was chosen to simulate the worst case scenario of a tumor with positive resection margins.

Intensity-modulated arc therapy was used. Coplanar plans used arcs in the transverse plane only, while non-coplanar plans used arcs in additional planes with slewing angles of ± 10°, ± 15° and ± 20°, relative to the transverse plane.

For each patient, a reference plan without slewing rotation (0°) was generated to compare with slewing angle plans. Slewing plans contained both the positive (e.g. +15°) and negative (e.g. -15°) slewing angles. For each plan, all possible beam incidences were provided in arcs to the optimization with the following specification: no incoming beam could pass through the arm, contralateral breast, or parts of the breast couch. Using a caudal beam incidence tilt, slewing resulted in a higher useful gantry angle range of the medial arcs. The final treatment plans contain arcs with caudal-medial incidence, with increasing gantry angle ranges (maximum over all patients) of 136°-179°, 132°-179°, 128°-179°, and 124°-179° for 0°, 10°, 15°, and 20° slewing, respectively. Plan optimization was performed using a multiple overlaying short arc VMAT technique which exploits optimal beam directions and reduces low dose spread to the OARs. VMAT planning tools, developed at Ghent University Hospital as extensions of the GRATIS treatment planning platform (Sherouse systems, Inc., Chapel Hill, NC, USA) are described elsewhere^22^. The number of final arcs, and their gantry span depend, patient-individually, on the optimizer. Intermediate and final dose calculations were performed using the convolution-superposition dose computation engine in Pinnacle 9.8. The objective was a median dose of 49.95 Gy in 15 fractions to the CTV boost, with 95% of the whole breast and lymphatic PTV volumes 5 mm underneath the skin to account for dose build-up (PTV_WBI_opt and PTV_Lnn_opt, respectively) covered by > 95% of the prescribed dose (40.05 Gy).

**Fig. 1:**
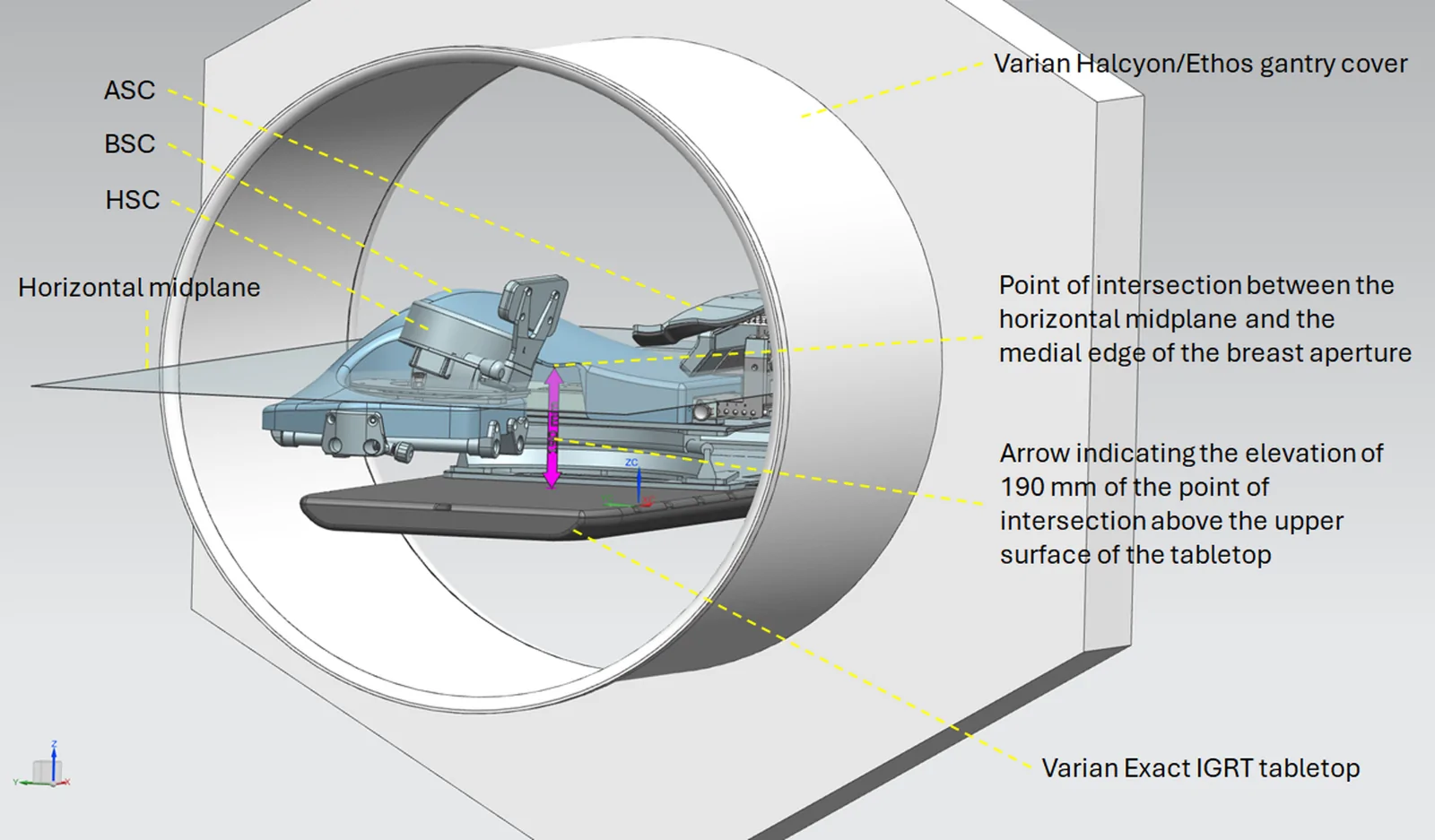
Reference position of the Prone Crawl Breast Couch inside the Varian Halcyon Ethos gantry cover. ASC: Arm Support Component. BSC: Body Support Component. HSC: Head Support Component. The centerline of the slewing axis passes through the tips of the magenta arrow. The distance between the upper surface of the Varian Exact IGRT tabletop and the medial edge of the breast aperture is 190 mm along the centerline of the slewing axis. The gantry cover has a diameter of 1 m.

In prone position, sufficient free space is needed above the breast couch for the patient’s body and below the aperture for the treated breast. The medial edge of the breast aperture was placed at the level of the horizontal midplane of the Halcyon/Ethos cylindrical gantry. Such placement leaves the space of the lower half-cylinder formed by the cylindrical gantry available for the treated breast, support structures of the prone breast couch and the Varian Exact IGRT tabletop. The space of the upper half-cylinder is available for the patient’s body and some parts of the prone breast couch (Fig. 1). This placement is further called *reference position* of the Prone Crawl Breast Couch inside the Varian Halcyon Ethos gantry cover.

Investigation of the free space between the cylindrical gantry covers of the Varian Halcyon/Ethos accelerator was performed using CAD models built in Siemens NX12. The maximum slewing angle of the prone crawl breast couch against the long axis of the Varian Exact IGRT tabletop was investigated in the reference position starting at ± 10° slewing and using ± 5° increments. Physical tests were done on the Varian Ethos linear accelerator at Ghent University Hospital.

Slewing was performed around a vertical axis through the isocenter intersecting the midline of the Varian Exact IGRT tabletop at index position H0 (slewing axis). Regarding the PCBC, the slewing axis was located at the medial edge of the breast aperture, 100 mm cranial to the caudal edge. By moving the Exact tabletop into the gantry, the slewing axis was positioned 560 mm cranially from the vertical lasers of Ethos. The Exact tabletop was lowered to position − 190 mm to reach the reference position for further measurements. The value of 190 mm is the distance from the surface of the Exact tabletop to the medial edge of the breast aperture at the position of the slewing axis (Fig. 1).

Statistical analysis was performed using R-studio version 4.2.1. (Posit PBC, Boston, Massachusetts, USA). For the target volumes the dose heterogeneity index (DHI) was calculated as follows: DHI = (D_2_-D_98_)/Dmean, with D_2_ being a measure for the maximum dose, D_98_ being a measure for the minimum dose and Dmean being the average of all dose values from D_0_ to D_100_. The DHI gives a measure of the spread of the radiation dose across the target volume, with a lower DHI representing a better dose homogeneity^14^. D_95_ was also recorded.

Shapiro’s test was used to test the normality assumption of the data. When data was normally distributed, repeated measures ANOVA was performed for each slewing angle. The sphericity of the data was automatically checked during this ANOVA. Mauchly’s test was internally used to assess the sphericity assumption. When the sphericity assumption was violated, the Greenhouse-Geisser correction was automatically applied. When statistically significant results were obtained from the repeated measures ANOVA, a T-test was performed for pairwise comparison, adjusted for multiple comparisons with the Bonferroni correction for the data that were normally distributed. When data were not normally distributed, a Friedman test was performed, followed by a pairwise comparison using the Wilcoxon signed rank test. The pairwise comparison using the Wilcoxon signed rank test was also adjusted for multiple comparisons using the Bonferroni correction. All significance levels were set to 0.05.

## Radiation risk model

Thirty-year mortality risk from radiation-induced cardiac injury, lung and esophageal cancer was calculated according to Aznar et al., Duane et al. and Taylor et al.^4,23–25^. The absolute risks of radiation-related mortality from heart disease and lung cancer for a 50-year-old (reference) patient were taken from Taylor et al.^4^. The absolute risk of radiation-induced cardiac mortality was estimated 0.075%/Gy and 0.3%/Gy mean heart dose for patients without and with cardiac risk factors, respectively. For radiation-induced lung cancer mortality, the risk was estimated 0.06%/Gy and 0.88%/Gy mean lung dose (both lungs) for patients who never smoked or continued smoking since adolescence, respectively. The method described by Duane et al.^25^ was used for calculating the absolute risk/Gy for mortality from esophageal cancer using the following figures: relative risk of 7.1%/Gy mean esophageal dose for cancer induction in non-smokers and 3-fold higher in smokers; long-term esophageal cancer mortality of 90% and absolute lifetime esophageal cancer risk for US female population at age 50 years = 233/100,000^26^. From these figures, absolute mortality-risk of radiation-induced esophageal cancer is estimated 0.015%/Gy and 0.045%/Gy for non-smoking and smoking patients, respectively. The rates/Gy, multiplied with the average mean heart or lung dose in Gy give an idea of the radiation-induced cardiac, lung and esophageal cancer mortality risk, respectively, for the different groups. Cumulative absolute risk of dying from heart disease, lung and/or esophageal cancer was calculated as 1-(1-P_h_)(1-P_l_)(1-P_e_) where P_h_, P_l_ and P_e_ are the risks to die from radiation induced heart disease, lung or esophageal cancer, respectively.

## Results

**Fig. 2:**
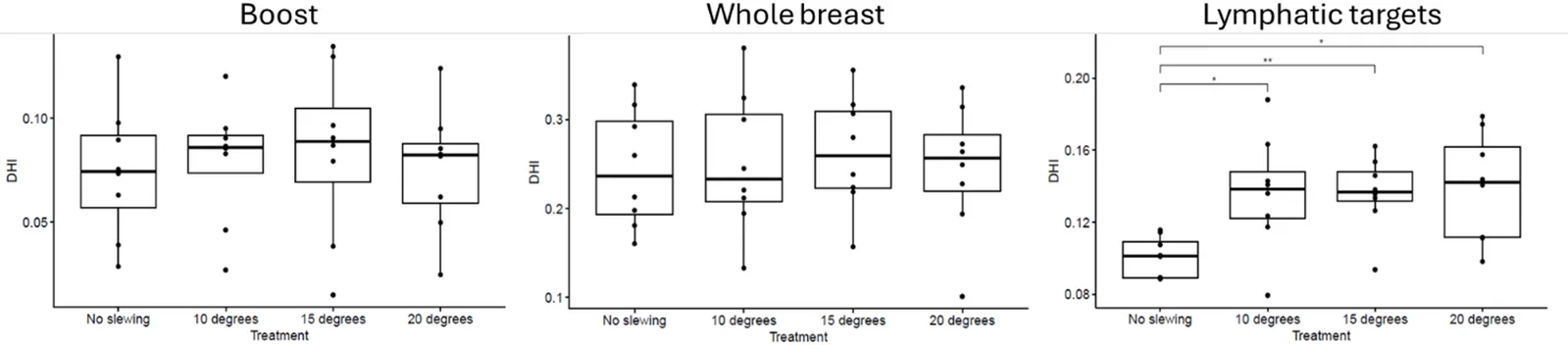
Overview of the comparison between the different slewing angle groups for each of the target volumes. DHI = dose heterogeneity index. The brackets with stars above them depict a statistically significant difference between the groups the bracket is located between. A single star depicts a p value below 0.05, a double star depicts a p value below 0.01.

For each patient, four treatment plans were made. A reference plan without slewing rotation (0°) was generated to compare with slewing angle plans 10°, 15° and 20°, respectively. Plan evaluation criteria were met for all plans. No significant differences in dose homogeneity were observed for whole breast or boost volume (Fig. 2). Dose homogeneity of the lymphatic target volumes was significantly better in coplanar plans. D_2_ and D_95_ is reported in Table 1.

**Table 1:** Doses reached by at least 95% of the volume of the structure (D_95_) and doses to a volume of 2% (D_2_) of the region of interest. Reported values are the averages ± the standard deviation of the averages. All values are in Gy.

PTV	Parameter	No slewing	10 degrees	15 degrees	20 degrees
Boost	D_95_	48.0 ± 1.0	47.9 ± 0.9	48.1 ± 1.0	48,0 ± 0.8
	D_2_	50.9 ± 0.3	51.2 ± 0.4	51.5 ± 0.8	51.1 ± 0.5
Whole breast	D_95_	38.7 ± 0.4	38.7 ± 0.4	38.6 ± 0.4	38.7 ± 0.4
	D_2_	47.8 ± 2.4	47.7 ± 2.5	48.1 ± 2.4	47.6 ± 2.7
Lymphatic targets	D_95_	38.7 ± 0.2	38.4 ± 0.3	38.5 ± 0.2	38.4 ± 0.2
	D_2_	42.1 ± 0.3	42.8 ± 0.5	42.9 ± 0.6	42.9 ± 0.7

Left lung doses (mean ± sd) were 4.85 ± 0.624 Gy, 3.90 ± 0.571 Gy, 3.30 ± 0.609 Gy and 3.21 ± 0.597 Gy for coplanar, ± 10°, ± 15° and ± 20° slewing plans, respectively (*p* < 0.001). Doses summed for both lungs were 2.49 ± 0.308 Gy, 2.00 ± 0.27 Gy, 1.72 ± 0.307 Gy and 1.69 ± 0.303 Gy for coplanar, ± 10°, ± 15° and ± 20° slewing plans, respectively (*p* < 0.001). Mean left or summed lung doses were not significantly different between ± 15° and ± 20° slewing plans. Both slewing plans resulted in approximately 30% mean lung dose reduction when compared to coplanar plans.

Heart doses (mean ± sd) were 1.21 ± 0.352 Gy, 0.969 ± 0.378 Gy, 0.908 ± 0.33 Gy and 0.96 ± 0.42 Gy for coplanar, ± 10°, ± 15° and ± 20° slewing plans, respectively (*p* < 0.001). Maximum LAD doses (D02 ± sd) were 9.34 ± 7.19 Gy, 5.73 ± 4.76 Gy, 5.33 ± 4.56 Gy and 5.71 ± 5.23 Gy for coplanar, ± 10°, ± 15° and ± 20° slewing plans, respectively (*p* < 0.001). Neither mean heart nor maximum LAD doses were significantly different between ± 10°, ± 15° and ± 20° slewing plans. On average, slewing plans resulted in approximately 20% and 35% decrease of mean heart and maximum LAD doses, respectively when compared to coplanar plans.

**Fig. 3:**
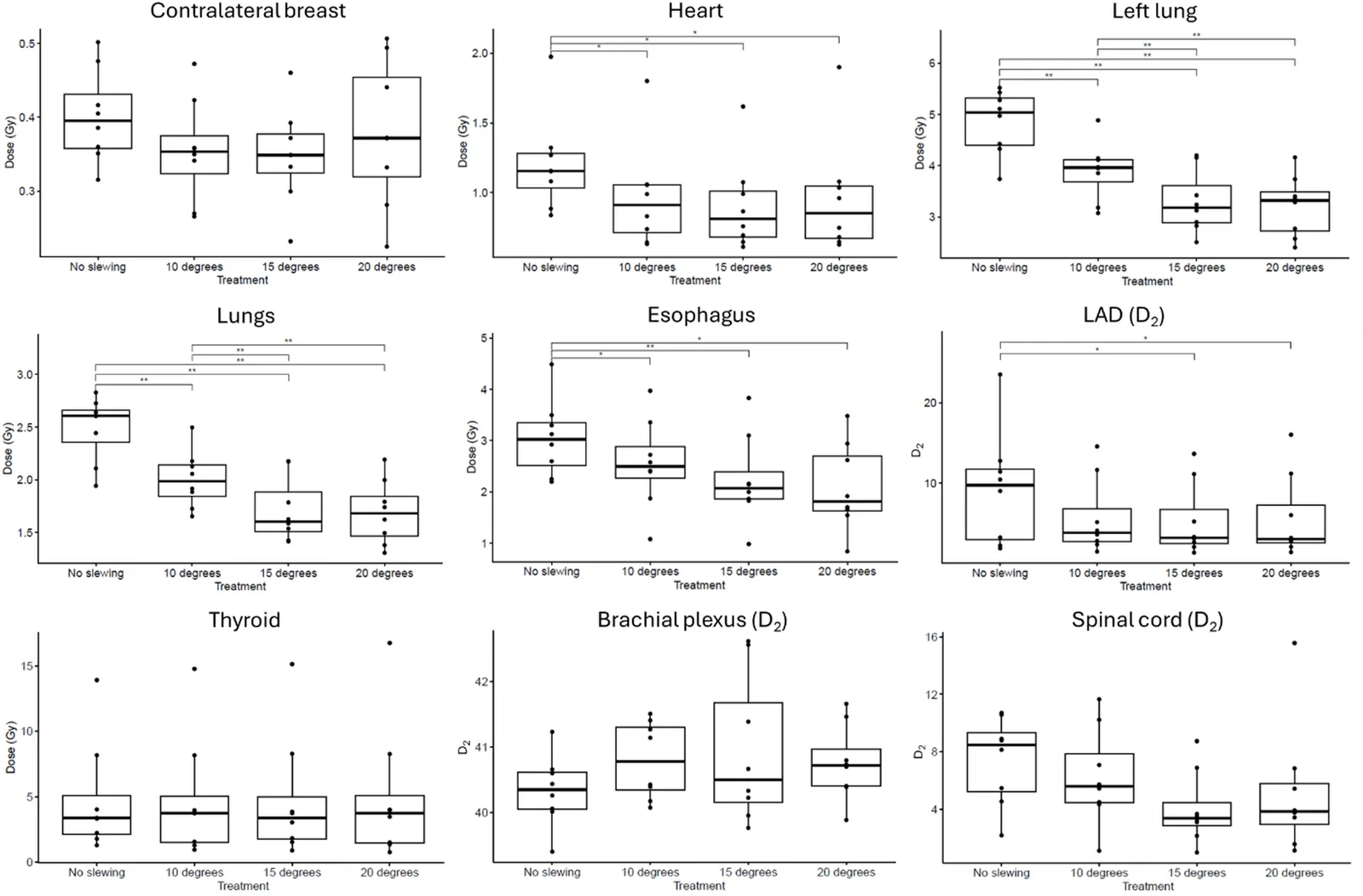
Comparison between the different slewing angle groups for organs-at-risk. The brackets with stars above them depict a statistically significant difference between the groups the bracket is located between. A single star depicts a p value below 0.05, a double star depicts a p value below 0.01.

Contralateral breast doses were less than 0.6 Gy for all individual plans and none of the differences were significant. Mean and maximum esophageal doses were significantly higher in the coplanar plans than in either of the slewing plans with no significant difference between ± 10°, ± 15° and ± 20° slewing plans. Maximum spinal cord doses were less than 16 Gy for all individual plans. No significant differences between coplanar and slewing plans were observed for mean thyroid or maximum brachial plexus doses. Comparisons between the coplanar and non-coplanar plans are shown in Table 2 and Fig. 3 for the organs at risk.

**Table 2:** Statistical comparison and mean dose and standard deviations for organs-at-risk. LAD = left anterior descending artery, D_2_ is a surrogate for the maximum dose, Gy = Gray. Statistically significant results are shown in bold text. P-values were corrected with a Bonferroni correction.

Organ at risk	Contralateral breast	Heart	Left lung	Lungs	Esophagus
Repeated measures ANOVA / Friedman test	F (3,21) = 1,72 / p = 0,19	X^2^ (3) = 16,35 / **p = 0,00096**	F (1.28, 8.95) = 36,13 / **p = 0,00012**	F (1.24, 8.66) = 35,48 / **p = 0,00016**	F (3, 21) = 10,06 / **p = 0,00026**
Individual comparisons					
0 vs 10 degrees slewing	p = 0,588	**p = 0,047**	**p = 0,009**	**p = 0,008**	**p = 0,047**
0 vs 15 degrees slewing	p = 0,053	**p = 0,047**	**p = 0,003**	**p = 0,003**	**p = 0,003**
0 vs 20 degrees slewing	p = 1	**p = 0,047**	**p = 0,002**	**p = 0,002**	**p = 0,029**
10 vs 15 degrees slewing	p = 1	p = 0,235	**p = 0,008**	**p = 0,007**	p = 0,592
10 vs 20 degrees slewing	p = 1	p = 1	**p = 0,001**	**p = 0,001**	p = 0,708
15 vs 20 degrees slewing	p = 1	p = 1	p = 1	p = 1	p = 1
Mean dose and standard deviation					
0 degrees slewing	0,401 ± 0,063 Gy	1,21 ± 0,352 Gy	4,85 ± 0,624 Gy	2,49 ± 0,308 Gy	3,05 ± 0,749 Gy
10 degrees slewing	0,355 ± 0,070 Gy	0,969 ± 0,378 Gy	3,90 ± 0,571 Gy	2,00 ± 0,27 Gy	2,55 ± 0,876 Gy
15 degrees slewing	0,348 ± 0,067 Gy	0,908 ± 0,33 Gy	3,30 ± 0,609 Gy	1,72 ± 0,307 Gy	2,24 ± 0,865 Gy
20 degrees slewing	0,378 ± 0,099 Gy	0,96 ± 0,42 Gy	3,21 ± 0,597 Gy	1,69 ± 0,303 Gy	2,09 ± 0,86 Gy

Organ at risk	LAD (D_2_)	Thyroid	Brachial plexus (D_2_)	Spinal cord (D_2_)
Repeated measures ANOVA / Friedman test	X^2^ (3) = 16,35 / **p = 0,00096**	F (1.35, 9.48) = 0,45 / p = 0,58	F (1.47, 10.26) = 1.48 / p = 0,27	F (3, 21) = 1,89 / p = 0,16
Individual comparisons				
0 vs 10 degrees slewing	p = 0,235	p = 1	p = 0,296	p = 1
0 vs 15 degrees slewing	**p = 0,047**	p = 1	p = 1	p = 0,122
0 vs 20 degrees slewing	**p = 0,047**	p = 1	p = 0,708	p = 1
10 vs 15 degrees slewing	p = 0,094	p = 1	p = 1	p = 0,599
10 vs 20 degrees slewing	p = 1	p = 1	p = 1	p = 1
15 vs 20 degrees slewing	p = 0,654	p = 1	p = 1	p = 1
Mean dose and standard deviation				
0 degrees slewing	9,34 ± 7,19 Gy	4,76 ± 4,28 Gy	40,3 ± 0,542 Gy	7,42 ± 3,04 Gy
10 degrees slewing	5,73 ± 4,76 Gy	4,76 ± 4,66 Gy	40,8 ± 0,591 Gy	6,26 ± 3,38 Gy
15 degrees slewing	5,33 ± 4,56 Gy	4,78 ± 4,78 Gy	40,9 ± 1,13 Gy	4,04 ± 2,54 Gy
20 degrees slewing	5,71 ± 5,23 Gy	5,00 ± 5,31 Gy	40,8 ± 0,579 Gy	5,21 ± 4,58 Gy

**Fig. 4:**
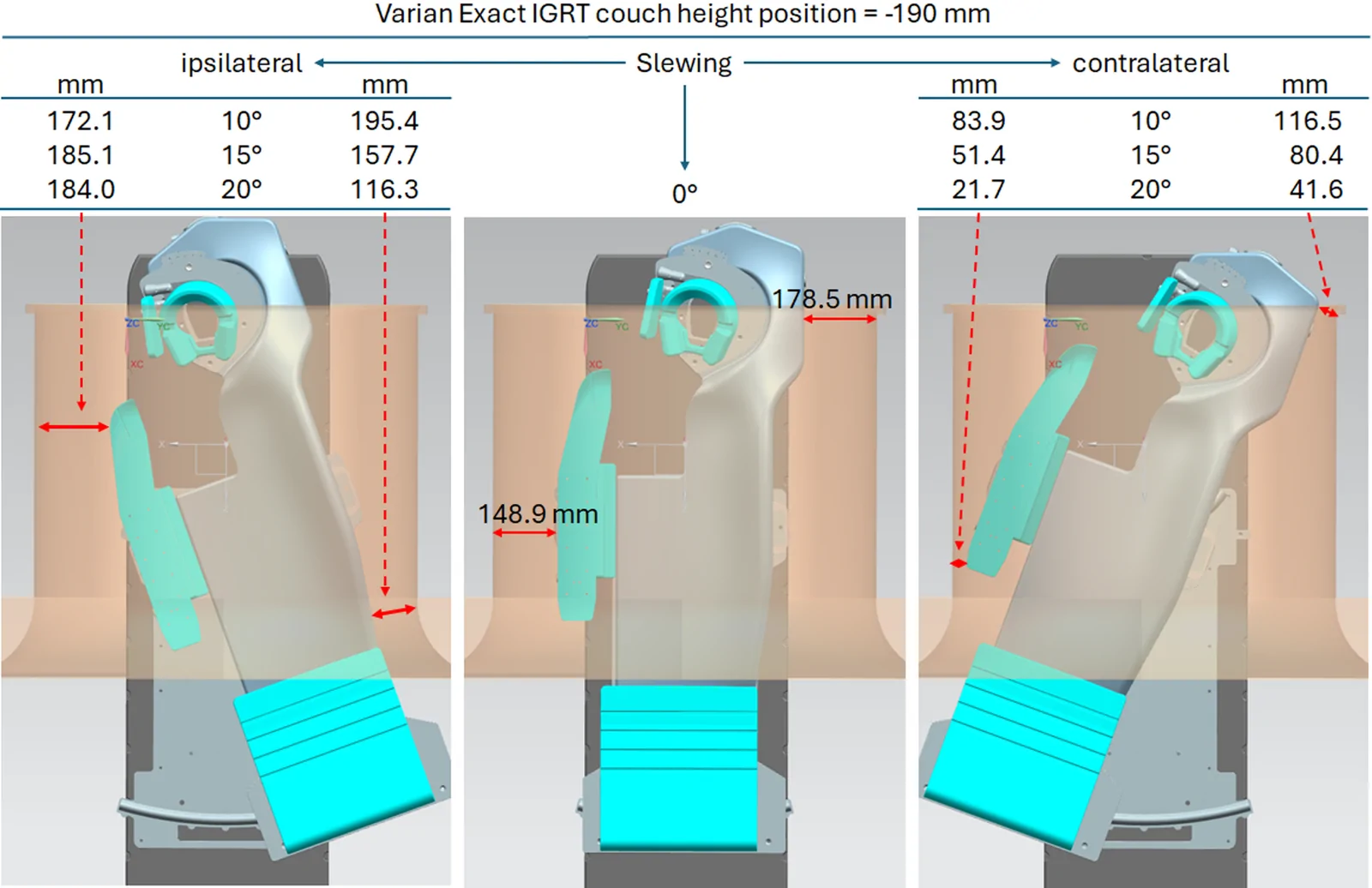
In-silico view from above of 0° and ± 20° slewing positions: ipsilateral = head towards the treated side, contralateral = head towards the non-treated side. Closest distances in millimeters between the PCBC and the cylindrical gantry cover of Varian Halcyon/Ethos accelerators at reference position of the Varian Exact IGRT couch. Gantry cover surface is the ocher-gray transparent drawing.

At the reference position, no collisions with the cylindrical gantry covers were observed for the ± 10°, ± 15° and ± 20° slewing positions of the PCBC. Figure 4 shows the regions of the PCBC which are nearest to collision. The lateral border of the arm support blade is closest to the gantry covers at the ipsilateral side. The caudal or cranial region of the border is closest at 10°, 15° and 20° contralateral or ipsilateral slewing, respectively. At the ipsilateral side of the PCBC, the closest regions to collision are cranially near the elbow or caudally near the knee support cushion, depending on the direction and magnitude of slewing (Fig. 4).

**Fig. 5:**
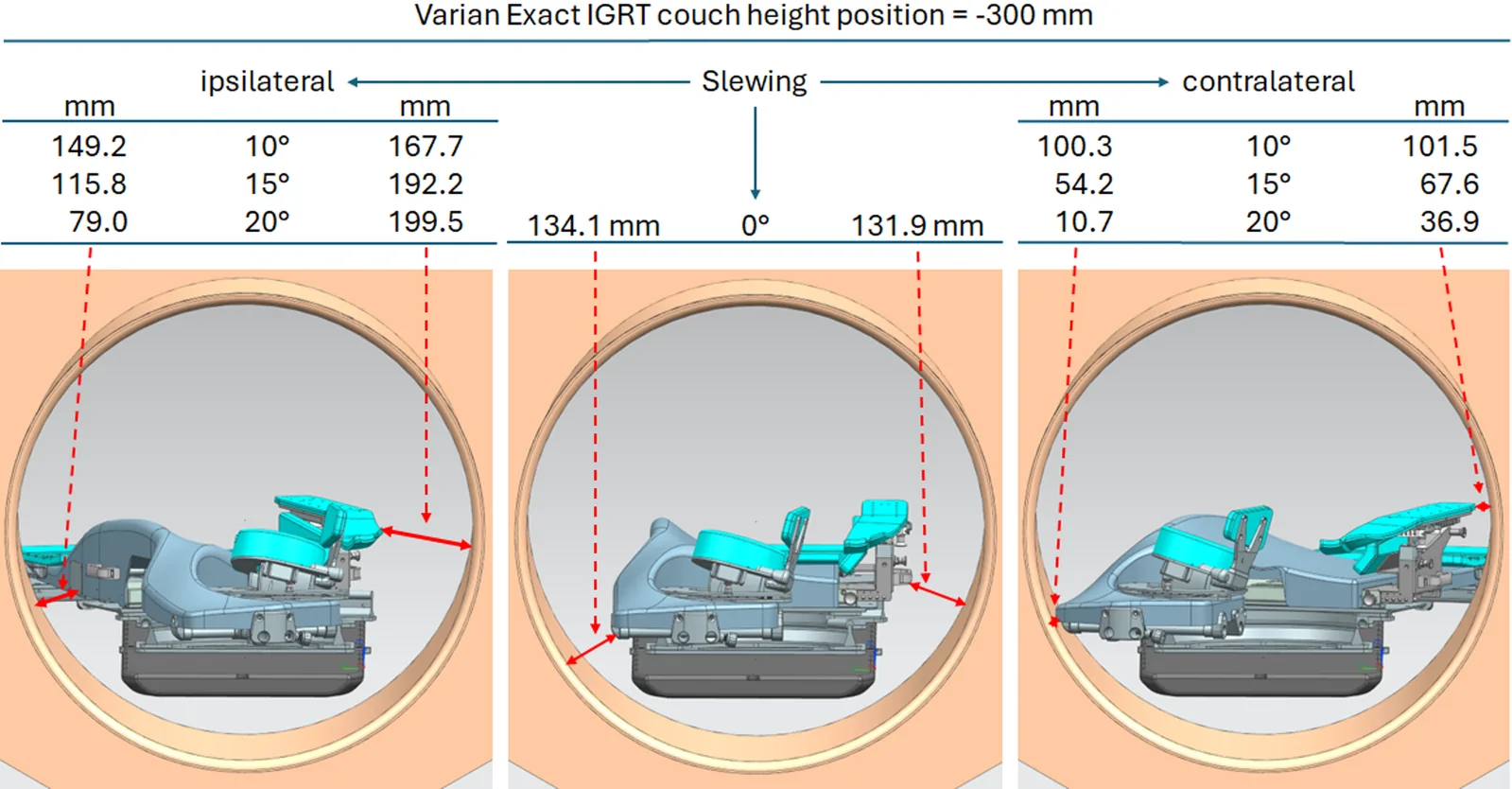
In-silico view through the Varian Halcyon/Ethos gantry aperture (ocher color) from behind. Slewing directions: ipsilateral = head towards the treated side, contralateral = head towards the non-treated side. Closest distances in millimeters between the PCBC and the cylindrical Gantry cover of Varian Halcyon/Ethos accelerators at reference position of the Varian Exact IGRT table assembly with the PCBC.

More free space for the patient’s body can be created by lowering the Varian Exact IGRT couch more than the − 190 mm value of the reference position. The technical limit of -360 mm below the isocenter can be reached except for the 20° contralateral slewing position for which collision between the gantry cover and the PCBC occurs at -320 mm table height. Measurements of free space are shown for table height − 300 mm in Fig. 5.

The slewing tests draw attention to two support surfaces of the PCBC. One region supports the upward positioned contralateral arm in the elbow region while the other supports the ipsilateral shoulder, arm and hand. Physical tests were performed to assess the minimum patient space defined as vertical projections from the upper surface of these PCBC regions to the upper half-cylinder formed by the gantry cover. A ± 10°, ± 15° and ± 20° indexed positioning aid was manufactured for accurate slewing of the PCBC on the Exact IGRT tabletop of the Varian Ethos accelerator at Ghent University Hospital. The patient free space is at least 190 mm for the contralateral elbow region. At the ipsilateral side, the minimum patient space is more than 190 mm for the shoulder and elbow and ≥ 116 mm for the ulnar side of the hand.

The space of 190 mm below the medial edge of the breast aperture may be insufficient for patients with pendulous breasts. The reproducibility of positioning is a challenge if the breast contacts the couch surface underneath, and the risk of skin toxicity increases by reduced build-up. The distance between the medial edge of the breast aperture and the Varian Exact IGRT couch surface can be increased by placing a spacer between the Varian Exact IGRT couch surface and the slewing pedestal of the PCBC. An interesting solution could be using a 60 mm spacer in combination with a -360 mm couch height position. The space for the treated breast increases to 250 mm while the free space for slewing remains identical to the values shown in Fig. 5.

## Discussion

Coplanar techniques are standard in adjuvant breast cancer radiotherapy except for partial breast irradiation. Non-coplanar techniques are of interest when it is desirable to reduce OAR dose beyond what is achievable with OAR-sparing techniques, like in adjuvant breast and lymph node radiotherapy. This study aimed to investigate if non-coplanar techniques can reduce radiation exposure to OARs on top of the dose-reductions already achieved using DIBH, prone positioning and advanced beam intensity-modulation.

Non-coplanar treatments usually consist of combinations of gantry and couch isocenter rotations. Couch isocenter rotations are not possible in a popular class of treatment machines like the Varian Halcyon/Ethos linear accelerators. However, non-coplanar treatments may still be possible in prone breast radiotherapy by slewing the prone breast couch against the couchtop of the linac. The possibilities of OAR-saving and technical limitations of such non-coplanar technique were investigated.

**Fig. 6:**
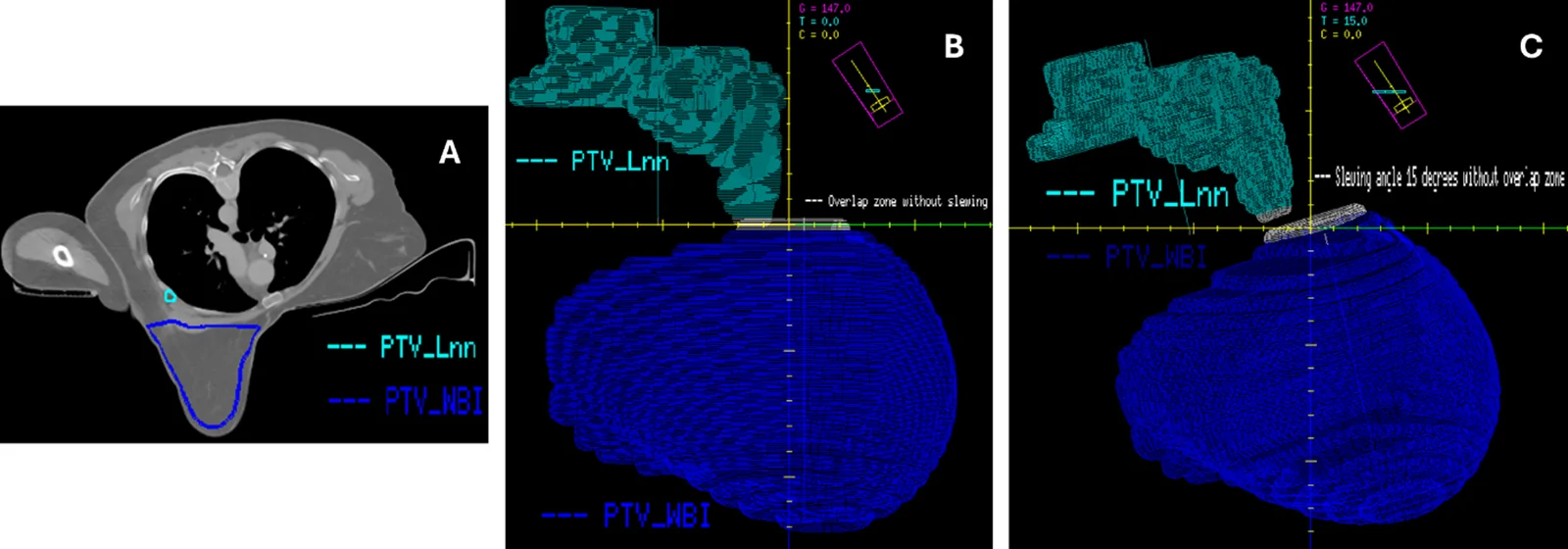
Visualization of whole breast (dark blue) and lymphatic target (light blue) PTVs. Panel A shows a CT slice displaying both the PTV for whole breast and lymphatic targets. Panel B shows the whole breast and lymphatic target PTVs in beam’s eye view in GRATIS^®^ treatment planning platform (Sherouse systems, Inc.,Chapel Hill, NC, USA) in a coplanar beam setup (no slewing). Note the overlap of the lymphatic target PTV and the whole breast PTV in this panel (white zone). Panel C shows the whole breast and lymphatic target PTVs in beam’s eye view, but with 15 degrees of slewing. Note that there is no overlap between both PTVs (the white zones do not touch) in panel C as a result of the 15 degree slewing angle. Overlap of the caudal part of PTV_Lnn and PTV_WBI in beam’s eye view in the coplanar plans (panel B) may have caused the better dose homogeneity in these plans, as compared to the 15 degree slewing angle plans. For this example, a gantry angle of 147 degrees was used. PTV_Lnn = planning target volume of lymphatic targets, PTV_WBI = planning target volume for whole breast irradiation.

When considering the target structures, there was no significant difference between the DHI for the whole breast and the DHI for the boost applied to the tumor bed when comparing the different slewing angles to the group without slewing. When looking at the results for the lymphatic target region, coplanar plans showed a significantly lower DHI, when compared to the groups with slewing. The lower DHI indicated a higher dose homogeneity across the lymphatic target area. The minimum doses as well as the maximum doses of the lymphatic target volume were closer to the prescription dose in coplanar plans than in slewing plans. A possible contributing factor to the better dose homogeneity in coplanar plans may be found in the overlap in beam’s eye view (BEV) of the cranial part of the PTV_WBI and the caudal part of the PTV_Lnn. In 6 out of 8 patients, there was at least one CT slice where parts of both PTV_WBI and PTV_Lnn are located (Fig. 6, panel A). Lower doses to the PTV_Lnn were most often located at the caudal part of this PTV. When using a coplanar beam setup, this caudal part of the PTV_Lnn can receive dose from beams targeting the PTV_WBI due to overlap in BEV (Fig. 6, panel B). When using slewing angles on the other hand, this caudal part of the PTV_Lnn may not overlap in BEV (Fig. 6, panel C), reducing the dose contribution of beams targeting the cranial part of PTV_WBI. Additional factors, such as different path lengths to PTV_Lnn parts may further have contributed to differences in DHI. The clinical impact of increased dose heterogeneity on lymph node targets is unclear.

The ipsilateral mean lung dose of 4.85 ± 0.6 Gy for coplanar plans was much lower than the 11.7 (SE 1.1) Gy that was reported for supine photon therapy for breast/chest wall + axillary/supraclavicular nodes, in a systemic review of lung doses published for the 2010–2015 period. This finding confirms the OAR sparing effect of the DIBH and the prone position compared to supine free breathing. At the same time, the mean ipsilateral lung dose in our study is comparable to that of supine proton doses for breast/chest wall + axillary/supraclavicular nodes reported in the same systematic review on lung doses 4.8 (SE 0.3)^23^. The current paper showed on average an additional 30% ipsilateral lung mean-dose reduction by using non-coplanar beam assemblies when compared to coplanar plans. Non-coplanar photon plans using ± 15° or ± 20° slewing in prone crawl position yield a ~ 3-fold reduction of mean ipsilateral lung dose as compared to supine photon therapy.

The mean heart dose of 1.21 ± 0.35 Gy for coplanar plans is slightly lower than the 2.24 ± 0.85 Gy from a similar study examining whole breast and lymph node irradiation (level II, III and IV) in prone position, but that study did not include breath hold techniques^12^. The mean heart dose of 1.21 ± 0.35 Gy is comparable to 1.3 (SE 0.1) Gy reported in a systematic review of heart doses published for the 2003–2013 period for left sided breast cancer radiotherapy not including the internal mammary chain. This figure relates to studies using breathing control, but these studies predominantly focused on breast-only radiotherapy^24^. Non-coplanar plans resulted in approximately 20% and 35% average decrease of mean heart and maximum LAD doses, respectively, when compared to coplanar plans. LAD maximum doses were approximately 40% smaller in non-coplanar plans with no significant differences between slewing angles.

The course of the mean heart dose in function of slewing angles suggests optimal slewing angles of about ± 15°, where a minimum heart dose of 0.908 ± 0.33 Gy is reached, about 0.06 Gy lower than for the ± 5° and ± 20° slewing plans. Speculatively, the higher heart doses for ± 20° compared to ± 15° slewing could originate from the increase of path lengths of the beam through the patient, increasing scattered dose contributions. Alternatively, there could be a reversal of proximity of beam apertures to the heart at slewing angles beyond ± 15°. LAD D_2_ and mean dose to contralateral breast is equally minimal at ± 15° slewing, while for mean lung and esophagus doses, a monotonic trend to lower values is observed from coplanar plans to ± 20° slewing.

The mean esophageal dose of 3.05 ± 0.75 Gy for coplanar plans is much lower than the doses for regional nodal breast cancer radiotherapy reported in a systematic review of esophageal doses published for the 2010–2022 period^25^. In this review, radiotherapy including the nodes delivered an average mean esophageal dose of 11.4 Gy. Non-coplanar photon plans using ± 10°, ± 15° or ± 20° slewing in prone crawl position yield a 15–30% reduction of mean esophageal dose as compared to coplanar plans.

Dose differences between coplanar and non-coplanar plans may be used to estimate average risk changes of mortality from radiation-related heart disease, lung and esophageal cancers. For a smoking 50-year-old reference patient, the risk of radiation-related death is estimated at 2.6%, 2.0%, 1.8% and 1.7% for coplanar, ± 10°, ± 15° and ± 20° slewing plans, respectively. The estimates are about 10-fold lower for a non-smoking reference patient with no other risk factors.

OAR dose differences between ± 15° and ± 20° slewing plans are numerically positive for some OARs and negative for others. Statistically significant differences are absent. Free space is larger for the smaller slewing angles. The center of gravity of the slewing PCBC-patient combination moves further lateral using the larger slewing angles which increases the technical challenges for building stiff, non-bending slewing mechanisms. Figure 4 shows that caudal parts of the PCBC move further laterally beyond the Varian tabletop at ± 20° than at ± 15° slewing, thereby limiting access to the patient in the gantry aperture. Considering clinical and technical parameters, ± 15° slewing angles seem to be the preferred settings for clinical trials.

Maximizing angular separation is considered important in non-coplanar radiotherapy, because it directly impacts dose conformity and normal tissue sparing. Striving for maximal angular separation may explain why data on small angular separation is scarce if not non-existing. Clearance issues may exist with conventional accelerators and the distance between the head of the linear accelerator and the isocenter seems to be a key factor^27^. Head-isocenter distances of 39.4–45 cm, that are typical for popular Varian and Elekta accelerators render non-coplanar techniques challenging, because of the need for plans with multiple isocenters. Using the PCBC, we have previously shown that non-coplanar intensity modulated radiation arc therapy (IMAT) with couch isocenter rotations up to 70° is feasible on conventional Elekta and Varian accelerators^14^. Practical constraints such as machine limitations, patient setup feasibility, and collision-avoidance with the treatment couch or gantry must be considered in applying couch isocenter rotations. The severity of these practical constraints is likely to decrease with less extreme couch isocenter rotations.

With the isocenter at 50 cm from the cylindrical covers that shield the head of the linac, Varian Halcyon/Ethos seems to be an attractive treatment machine for prone breast radiotherapy. The central panels (slewing 0°) in Figs. 4 and 5 give an idea of the available space for the prone positioned patient in coplanar treatments. The Halcyon/Ethos has a 28 × 28cm^2^ field size that limits the possible range of isocenter locations in patients with pendulous breast and/or large body size, or may even require the use of more than one isocenter. In this study, all PTV sizes were below 24.5 cm in any direction and allow treatment with the Halcyon linac in a single isocenter.

Currently, couch rotations are not supported by Varian Halcyon/Ethos accelerators. Hard- and software limitations exist for implementing non-coplanar prone techniques with Halcyon/Ethos linear accelerators. The breast and lymph node regions must be placed above the Varian couchtop, because the Halcyon/Ethos software does not allow irradiation of treatment volumes extending beyond the couchtop’s cranial edge. This means that the treated breast and the patient’s thorax are stacked above the couchtop. In practice, the couchtop’s upper surface must be placed below the isocenter to avoid collisions of the treated breast with the couch and of the patient’s back with the cylindrical gantry cover. Lowering the Varian couchtop inside the lower half of the gantry cover progressively restricts its freedom of rotation around a vertical axis. With the couchtop at -190 mm height (as shown in Figs. 1 and 4) and fully inserted in the cylindrical gantry cover, collision between couchtop and cover would occur at approximately ± 20° rotation around a vertical axis through the isocenter. At -300 mm couchtop height (as shown in Fig. 5), collision would occur at approximately ± 10° rotation of the Varian couchtop, assuming the couchtop could rotate. By slewing the breast board against the linac couchtop, ± 20° rotation of the breast board is possible at -190 mm and − 300 mm couchtop heights.

The Varian Eclipse planning system has hardcoded restrictions for Halcyon/Ethos which make planning of slewing techniques challenging if not impossible. This study suggests that such restrictions are associated with significantly larger OAR-doses than would be achievable if slewing angles could be planned. Eclipse does not allow comparative planning in the Halcyon/Ethos environment. Therefore, comparative planning was performed on a general-purpose planning system with 3D capabilities regarding beam directions. It should be kept in mind that set up reproducibility with slewing, intrafraction motion during DIBH and staff training should also be considered for the possible future implementation and further validation of the technique described in this manuscript. A further limitation of this study is that the beam model that we used was a single-bank MLC and not the dual-bank MLC of Halcyon/Ethos. However, both systems have similar capabilities regarding complex intensity profiles for IMRT and VMAT.

## Conclusion

Heart- and lung-sparing techniques like DIBH and prone positioning represent a major leap forward in adjuvant breast cancer radiotherapy over the last decades. Except for partial breast irradiation, coplanar techniques are standard routine due to their simplicity and efficiency. This study shows that non-coplanar techniques can reduce radiation exposure to OARs on top of the dose-reductions already achieved using DIBH, prone positioning and advanced beam intensity-modulation. Adding beams in planes with ± 15° angular separation with the transverse plane yielded > 20% mean dose reductions simultaneously to heart, lungs and esophagus in breast and regional lymph node irradiation. This study also showed that plans with ± 15° angular separation with the transverse plane could be made feasible in the future on linacs that lack couch isocenter rotations like Varian Halcyon/Ethos. Help from the manufacturer is needed to relieve restrictions that prohibit non-coplanar treatments.

## Data Availability

The datasets generated during and/or analyzed during the current study are available from the corresponding author on reasonable request.
